# Assessment of Risk Factors for the Occurrence of a High-Output Ileostomy

**DOI:** 10.3389/fsurg.2021.642288

**Published:** 2021-05-21

**Authors:** Claudia Seifarth, Leonard N. Augustin, Kai S. Lehmann, Andrea Stroux, Johannes C. Lauscher, Martin E. Kreis, Christoph Holmer

**Affiliations:** ^1^Department of General-, Visceral- and Vascular Surgery, Charité – Universitätsmedizin Berlin, corporate member of Freie Universität Berlin, Humboldt-Universität zu Berlin, and Berlin Institute of Health, Berlin, Germany; ^2^Institute for Biometry and Clinical Epidemiology, Charité – Universitätsmedizin Berlin, corporate member of Freie Universität Berlin, Humboldt-Universität zu Berlin, and Berlin Institute of Health, Berlin, Germany; ^3^Department of General and Visceral Surgery, St. Joseph Hospital, Berlin, Germany

**Keywords:** high-output stoma, risk factors, ileostomy, IBD, colon cancer

## Abstract

**Background:** Ileostomy is often required in patients undergoing bowel resections for inflammatory bowel diseases (IBD), colorectal cancer, and emergencies. Unfortunately, some patients develop a high-output stoma (HOS). This condition affects homeostasis and may be life threatening. We aimed to identify possible risk factors for the development of HOS.

**Patients and methods:** From 2012 to 2018, 296 patients who underwent ileostomy at Charité – Universitätsmedizin Berlin, Campus Benjamin Franklin were retrospectively analyzed. Emergency operations were included. Diverting ileostomy, end ileostomies and anastomotic stomata with at least part ileum, were examined. HOS was defined as stoma output of more than 1,000 mL per day for more than 3 days. Univariate and multivariable analyses were used to detect potential risk factors for the development of HOS.

**Results:** 41 of 296 patients developed HOS (13.9%). Mortality was 0.3%. In the univariate analysis, age (*p* < 0.002), diagnosis (Crohn's disease, *p* = 0.005), arterial hypertension (*p* = 0.023), surgical procedure (right-sided colectomy, small bowel resection, *p* < 0.001), open technique (*p* < 0.002), emergencies (*p* = 0.014), and anastomotic ileostomy (*p* < 0.001) were identified as risk factors. In the multivariable logistic regression, older age, diagnosis (Crohn's disease) and surgical procedure (right-sided colectomy, separate ileostomy, small bowel resection) remained significant risk factors.

**Conclusion:** The occurrence of HOS is a relevant problem after ileostomy. The identification of risk factors for a high-output may be helpful for monitoring, early diagnosis and initiation of therapy as well as in the planning of close follow-up care.

## Introduction

Temporary or permanent ileostomies are used in a variety of diseases, e.g., in the operative therapy of inflammatory bowel diseases (IBD) such as Crohn's disease (CD) and ulcerative colitis (UC) or in colorectal cancer. In the post-operative course, several complications can occur which are related to an ileostomy. In addition to local complications such as retraction or prolapse of the stoma, dermatitis and erythema, leakage, or obstruction, a high-output stoma (HOS) can develop (1). A high-output situation arises when the fluid loss through the ileostomy is higher than the compensation mechanisms of the patient. As a result, dehydration, electrolyte shift, acute kidney failure, deep vein thrombosis, pulmonary embolism, and even death may occur (2). The resulting complicated course may require longer hospital stays and repeated hospital stays. Although HOS is a known complication with an incidence of up to 18% (3), perioperative risk factors for the development of HOS are not yet sufficiently known, despite recent studies on this topic (4, 5).

There is a wide range of definitions for HOS in the literature. Most authors refer to the cumulative stoma output within 24 h as a diagnostic criterion. Previous literature refers to different stoma output values such as >1,500 mL per day (5), >2,000 mL per day (1), at least once >2,000 mL/day, but >1,000 mL/day for at least 3 days (4) or 2,000 mL/day for at least 3 days (6, 7).

A known cause for development of a HOS is the length of the remaining sections of the small intestine. In addition to high electrolyte losses, a negative fluid and caloric balance may be observed in patients with a jejunum that remains <100 cm orally from the stoma (8, 9). In ileostomy, the pure length of the remaining ileum with preserved jejunum was not described as a risk factor for the development of HOS. There is evidence that post-operative gastrointestinal infections, e.g., Clostridium difficile enteritis, a partial or intermittent post-operative obstruction of the intestinal lumen, sudden post-operative discontinuation of existing medication with glucocorticoids or opioids, use of prokinetics or a relapse of an existing underlying disease (e.g., Crohn's disease, radiation enteritis) can contribute to HOS (6). With regard to the choloresorptive function of the terminal ileum, chologenic diarrhea in bile acid malabsorption syndrome was discussed as a trigger for increased stool frequency after an ileostomy, especially if <50 cm of the terminal ileum was resected. In this case, the malabsorption of bile acids clearly outweighs that of water (10). The higher intraluminal content of bile acids can contribute to the development of watery diarrhea in the ileum through an osmotic, active secretory, and cytotoxic effect. This effect probably correlates with the length of the resected terminal ileum. From a resection size of about 100 cm terminal ileum, the increased excretion of bile acids also outweighs the compensation capacity of liver synthesis and the bile acid pool is exhausted. The latter can also lead to severe diarrhea and steatorrhea by reducing the absorption of free fatty acids and thus water (11).

Overall, not much is known about the pathogenesis and etiology of HOS. However, it is a potentially dangerous post-operative complication that needs a better understanding to be avoided or to be treated properly. The aim of this study was to identify possible risk factors for the development of HOS.

## Patients and Methods

### Patients

All patients of Charité – Universitätsmedizin Berlin, Department of Surgery, Campus Benjamin Franklin that underwent an ileostomy between January 1st, 2012 to April 1st, 2018 were included consecutively in this study. The study was carried out retrospectively based on patient records. The following two groups were evaluated:

A: Patients with high-output ileostomy.
B: Patients without high-output ileostomy.

All planned and unplanned operations were included. Only diverting and terminal ileostomies and anastomotic stomata, which consist of at least part of the ileum (12), were included.

### Methods

In this retrospective analysis, data were collected from the hospital's in-house computer system. The primary outcome parameter was post-operative high-output ileostomy. All planned and unplanned operations were included. The following variables were used to assess perioperative characteristics: age, body mass index (BMI), gender, American Society of Anesthesiologists (ASA) score, diagnosis, secondary diagnoses, immunosuppressant medication, nicotine abuse, surgical procedure and technique, type of ileostomy, length of surgery. Nicotine abuse: since data about pack years were unreliable, smokers were divided into active smokers (nicotine abuse within 1 month prior to the operation) and non-smokers (no or former nicotine abuse). Post-operative outcomes are reported with these variables: complication rate (classified by Clavien-Dindo), HOS, mortality during inpatient stay.

### Definition of High-Output Stoma

HOS was defined as stoma output of more than 1,000 mL per day for more than three consecutive days, which could not be regulated by dietary measures and sufficient hydration.

### Statistics

Normality was tested with the Shapiro-Wilk test. Since most variables showed no normal distribution, continuous variables are displayed as median (minimum-maximum) and categorical variables are displayed as count (percentage). Accordingly, for univariable group comparison, logistic regression (Odds ratio, CI 95%) with *p* < 0.05 considered as statistically significant.

Multivariable logistic regression analysis was performed to assess potential risk factors for the development of HOS. Only significant variables from the univariable analysis were entered into the regression model.

For checking a linear relationship between the variable and the logit of the response variable the Box-Tidwell test was used. All statistical analyses were performed with SPSS Statistics Software 25.0 (IBM, Armonk, NY, USA).

### Ethics

The study protocol was approved by the ethics committee of the Charité - Universitätsmedizin Berlin (EA2/088/18).

## Results

### Demographics

Between January 1, 2012 and April 1, 2018, 296 patients underwent ileostomy because of ulcerative colitis (UC: 33.4%), Crohn's disease (CD: 21.6%), carcinoma (24.7%), and other diagnoses (20.3%). Emergency procedures accounted for 33.8% of all operations. The median age at the time of operation was 50 years (15–83). 42.2% of the patients were female. BMI was 23.9 m^2^/kg (13.8-45.1) and most patients were categorized ASA 1 and 2 (78.7%). 41 of 296 patients developed a HOS (13.9%). The mortality rate was 0.3% (Table 1).

**Table 1:** Demographics.

**All patients**	***n* = 296**
**Diagnosis**	
Ulcerative colitis	99 (33.4)
Crohn's disease	64 (21.6)
Carcinoma	73 (24.7)
Varia	60 (20.3)
Emergency procedures	100 (33.8)
Age, years	50(15–83)
**Sex**	
Female	125 (42.2)
Male	171 (57.8)
BMI, kg/m^2^	23.9 (13.8–45.1)
**ASA**	
1, 2	233 (78.7)
3, 4	63 (21.3)
HOS	41 (13.9)
Mortality	1 (0.3)

### Risk Factors for High-Output Stoma (Univariate Analysis)

Patient characteristics in the “HOS” and “No HOS” groups were balanced in respect to BMI (*p* = 0.909), sex (*p* = 0.566), and ASA (*p* = 0.083, Table 2). Patients with HOS were older than patients without HOS (57 vs. 48 years, *p* = 0.002). The analysis of risk factors for the development of a HOS showed significant differences for the diagnosis on which ileostomy was based. Patients with Crohn's disease (CD) developed HOS significantly more often than patients with UC, carcinoma or other diagnoses (CD: 23.4%, UC: 7.1%, carcinoma: 9.6%, varia: 20%; *p* = 0.012). Apart from arterial hypertension (*p* = 0.023), no other secondary diagnoses (COPD, coronary artery disease, chronic kidney failure, heart failure, diabetes mellitus) or immunosuppressive drugs or smoking showed influence on the development of HOS.

**Table 2:** Patient-specific risk factors, univariate analysis.

	**Ileostomy (*****n*** **= 296)**		**Missing**	**OR (95 % CI)**	***P*-value**
	**No HOS (*n* = 255)**	**HOS (*n* = 41)**			
Age, years	48 (15–83)	57 (30–82)	0	1.032 (1.011–1.053)	0.002
BMI, kg/m^2^	24.1 (13.8–45.1)	23.4 (14.3–37.4)	21	0.996 (0.993-1.065)	0.909
Sex					
Male	149 (87.1)	22 (12.9)	0	-	0.566
Female	106 (84.8)	19 (15.2)		1.214 (0.626-2.355)	
ASA					
1, 2	205 (88)	28 (12)	0	-	0.083
3, 4	50 (79.4)	13 (20.6)		1.904 (0.920-3.938)	
Diagnosis					
Ulcerative colitis	92 (92.9)	7 (7.1)	0	-	0.012
Crohn's disease	49 (76.6)	15 (23.4)		4.023 (1.538-10.526)	0.005
Carcinoma	66 (90.4)	7 (9.6)		1.394 (0.467-4.164)	0.552
Other	48 (80)	12 (20)		3.286 (1.214-8.890)	0.019
Arterial hypertension	67 (78.8)	18 (21.2)	0	2.196 (1.116-4.321)	0.023
COPD	8 (80.0)	2 (20.0)	0	1.583 (0.324-7.732)	0.570
Coronary artery disease	19 (86.4)	3 (13.6)	0	0.981 (0.277-3.474)	0.976
Chronic renal failure	12 (85.7)	2 (14.3)	0	1.038 (0.224-4.818)	0.962
Heart failure	8 (88.9)	1 (11.1)	0	0.772 (0.094-6.338)	0.810
Diabetes mellitus	21 (77.8)	6 (22.2)	0	1.910 (0.721-5.061)	0.193
Immunsuppression			0		0.193
No	212 (87.6)	30 (12.4)		-	
Glucocorticoids	25 (86.2)	4 (13.8)		1.131 (0.386-3.474)	0.830
Other	10 (76.9)	3 (23.1)		2.120 (0.552-8.142)	0.274
Multiple (≥2)	8 (66.7)	4 (33.3)		3.533 (1.003-12.452)	0.050
Nicotine abuse			15 (5.1)		0.096
Active	46 (79.3)	12 (21.6)		-	
Non smoker	196 (87.9)	27 (12.1)		1.894 (0.893-4.017)	

*Continuous variables as age and BMI are median (minimum-maximum). Categorical variables are count (percentage). OR, odds ratio; CI, confidence interval*.

Regarding surgical procedures, small bowel resection was associated with HOS more often than any other operation (43.5%, *p* < 0.001, Table 3). After ileo-pouch anal anastomosis (IPAA), right colectomy, and sole ileostomy, patients developed HOS more often compared to left colectomy. Patients with open surgery showed more HOS compared to minimally invasive procedures (20 vs. 6.9%, *p* = 0.002). After emergency cases, HOS was significantly more common than after elective surgery (*p* = 0.014). Patients with an anastomotic stoma showed more HOS than patients with loop or end ileostomy (*p* < 0.001). Length of surgery and post-operative morbidity had no influence on HOS.

**Table 3:** Operation specific risk factors for HOS.

	**Ileostomy (*****n*** **= 296)**		**OR (95 % CI)**	***P*-value**
	**No HOS** **(*n* = 255)**	**HOS** **(*n* = 41)**		
Surgical procedure				<0.001
Left-sided colectomy	79 (97.5)	2 (2.5)	-	
Ileo pouch-anal anastomosis (IPAA)	47 (95.9)	2 (4.1)	7.473 (1.480-37.733)	0.015
Procto-colectomy	37 (84.1)	7 (15.9)	1.681 (0.229-12.333)	0.610
Right-sided colectomy	36 (76.6)	11 (23.4)	12.069 (2.543-57.283)	0.002
Sole Ileostomy	43 (82.7)	9 (17.3)	8.267 (1.709-40.000)	0.009
Small bowel resection	13 (56.5)	10 (43.5)	30.385 (5.968-154.704)	0.000
Surgical technique				0.002
Open	128 (80.0)	32 (20.0)	-	
Minimal-invasive	122 (93.1)	9 (6.9)	0.295 (0.135-0.644)	
Missing	5 (100)	0	-	
Surgical priority				0.014
Emergency	69 (78.4)	19 (21.6)	-	
Elective	186 (89.4)	22 (10.6)	0.430 (0.219-0.843)	
Type of ileostomy				<0.001
Loop	202 (88.6)	26 (11.4)	-	
End	48 (88.9)	6 (11.1)	0.971 (0.379-2.491)	0.951
Anastomotic	5 (35.7)	9 (64.3)	13.985 (4.353-44.923)	<0.001
Length of surgery, min	233 (32-761)	205 (77-496)	0.999 (0.996-1.002)	0.404
Clavien-Dindo				0.069
1, 2	200 (88.1)	27 (11.9)	-	
3-5	54 (79.4)	14 (20.6)	1.920 (0.942-3.914)	0.072

*Continuous variables as age and BMI are median (minimum-maximum). Categorical variables are count (percentage). OR, odds ratio; CI: confidence interval*.

### Risk Factors for High-Output Stoma (Multivariate Analysis)

All significant variables from the univariate analysis were included in the multivariable logistic regression model (Table 4). The development of HOS was independently associated with older age. Patients with Crohn's disease were at higher risk for HOS compared to patients with ulcerative colitis or patients with carcinoma or other diagnoses. Furthermore, the kind of surgical procedure was associated with HOS. Especially right-sides colectomies including ileo pouch-anal anastomosis (IPAA), and ileocoecal resection as well as sole ileostomy and small bowel resections were at higher risk for HOS.

**Table 4:** Multivariable logistic regression of risk factors for the development of HOS.

	**OR (95 % CI)**	***P*-value**
Age	1.055 (1.025-1.086)	0.001
Diagnosis		0.035
Ulcerative colitis (UC)	–	–
(reference)		
Crohn's disease (CD)	11.326 (1.799-71.289)	0.010
Carcinoma	2.555 (0.396-16.478)	0.324
Other	4.026 (0.593-27.305)	0.154
Surgical procedure		0.008
Left-sided colectomy (reference)	–	–
Ileo pouch-anal anastomosis	67.304 (7.006-646.581)	<0.001
(IPAA)		
Procto-colectomy	9.970 (0.803-123.755)	0.074
Right-sided colectomy	8.559 (1.483-49.380)	0.016
Sole Ileostomy	8.303 (1.652-41.728)	0.010
Small bowel resection	11.910 (1.718-82.580)	0.012

*OR, odds ratio; CI, confidence interval*.

## Discussion

The high-output ileostomy is one of the complications after ileostomy and can result in longer and repeated hospital stays and a reduced quality of life for the patient. Perioperative risk factors for developing HOS have not yet been adequately studied. In this study, we were able to identify age, diagnosis (Crohn's disease) and surgical procedure (right-sided colectomy, separate ileostomy, small bowel resection) as significant risk factors.

### The Incidence of HOS

The incidence of high-output ileostomy was 13.9% in our study. Only a few studies concentrated on ileostomies for the development of HOS and the incidences for HOS were often significantly higher. Takeda et al. found an incidence of 23.8% in their collective of 164 patients with colorectal cancer (13). Baker et al. reported an incidence of 16%, but they did not differentiate between the ileostomy and jejunostomy (>200 cm or <200 cm small intestine orally of the stoma) (6). The occurrence of HOS was inversely correlated to the length of the small intestine proximate to the stoma in the former study, i.e., shorter bowel correlated with higher incidence of HOS. Since we specifically analyzed ileostomies, studies that did not distinguish between ileostomy and jejunostomy or included colostomies were difficult to compare with our work. In contrast to other studies, we did not limit the diagnosis that led to ileostomy formation to certain diseases but included all IBD patients as well as carcinoma patients and patients with other diagnoses. In the work of Hara et al. from 2020, 81.2% of the patients had colorectal cancer and the incidence of HOS was 31% (5). A slightly lower incidence of 18% was reported by Park et al. in their study on the occurrence of post-operative complications after ileostomy in 74 patients with ulcerative colitis only (3). A Dutch study by Bakx et al. showed an incidence of HOS of only 4.3% in 69 patients. However, only temporary ileostomies were included here, and patients with anastomotic leakage and patients with inflammatory bowel diseases were excluded (14).

### Risk Factors for HOS

In the present work, numerous parameters were examined with regard to a potential role as risk factors in the development of HOS. Our analysis showed that higher age, the presence of Crohn's disease, right-sided colon surgery including proctocolectomies and small bowel resections significantly increased the risk for HOS.

Older patient age was already included in risk analyses of previous works on HOS. However, there are no uniform results, since some studies also describe younger age as a risk factor for HOS (4, 5, 7, 13). In contrast to our work, only patients with colorectal cancer were examined in the aforementioned studies. In line with our results, Justiniano et al. also showed that older age correlated significantly with a higher rate of re-hospitalization due to dehydration regardless of the surgical diagnosis (15). In summary, higher patient age could not be clearly identified as a favorable or an unfavorable factor in previous studies. The present work makes an important contribution to the discussion insofar as a high number of cases was included and no pre-selection of certain subgroups was carried out.

The surgical diagnosis was another risk factor for the development of a HOS in our study. CD patients developed a HOS significantly more often than patients with ulcerative colitis or colorectal cancer. The majority of previously published studies focused on patients with colorectal cancer. To date, there is little knowledge available to compare the variety of diseases leading to surgery and their role as potential independent risk factors for HOS. It is known from case reports that HOS occurs in patients with CD (16), especially for patients with extensive small bowel resections and jejunostomy. In our work, we excluded patients with jejunostomies and only examined ileostomies. A possible reason for the higher rate of HOS in CD is the resection of parts of the ileum that is frequently necessary. CD is associated with a disturbance in the intestinal permeability or barrier. Reduced tight junction proteins lead to increased cellular permeability even when there is no macroscopic lesion of the intestinal mucosa (17). Altered gut microbiota also play a role in complex interaction with intestinal epithelial cells (18). In addition, traumatic tissue damage can promote the release of further pro-inflammatory cytokines stimulated by increased TNF-α levels in the serum (19, 20). The resulting impairment of the intestinal permeability/barrier can lead to HOS.

The type of surgery was identified as another risk factor for HOS in our work. In particular, right-sided colectomies (including Ileocecal resections), (procto-) colectomies and isolated ileum segment resections, which were usually associated with a protective ileostomy or an anastomotic stoma, were identified as factors. It is known that shortening the small intestine with the placement of a more proximal ileostomy can lead to HOS (8, 21). This is based on a disturbance in intestinal permeability that can occur after abdominal surgery (22). Here, cytokines such as TNF-α, which are released in the event of tissue damage, play a central role. In IBD, a disturbance in intestinal permeability is already pathognomonic in the early stages of the disease (17). If there is an intestinal resection, tissue damage increases and an increased cytokine cascade with increased disruption of the intestinal barrier resulting in HOS, is conceivable.

Another approach to the development of HOS after resection of the terminal ileum results from the observation of the neuropeptide metabolism. Above all, neuropeptide YY (PYY), released in the terminal ileum, has an appetite-reducing effect by inhibiting gastrointestinal motility and stimulating electrolyte secretion (23). After ileum resections, the neuropeptide metabolism initially gets out of balance. Postprandial feedback is eliminated, intestinal hypermotility and hypersecretion of electrolytes occur. The role of PYY has already been discussed in the pathogenesis of patients with short bowel syndrome (24), but has so far been less well-known in HOS.

## Limitations

A limitation of our study was the retrospective study design. Despite the comparatively high number of almost 300 patients in one center, we could not assess all potential risk factors for the development of HOS. Nicotine, for example, is known for its negative impact on post-operative outcome such as wound or anastomotic healing and the relapse rate of Crohn's disease. However, the data for nicotine abuse were missing in 5%. A conclusive result regarding the significance could not be made.

## Conclusion

Old age, Crohn's disease and right-sides colectomies including ileo pouch-anal anastomosis (IPAA) were identified as risk factors for a post-operative high output stoma. In these patients, special attention should be paid to the ostomy output in order to identify and treat HOS early.

## Data Availability Statement

The original contributions presented in the study are included in the article/supplementary material, further inquiries can be directed to the corresponding author/s.

## Ethics Statement

The studies involving human participants were reviewed and approved by ethics committee of the Charité - Universitätsmedizin Berlin (EA2/088/18). Written informed consent for participation was not required for this study in accordance with the national legislation and the institutional requirements.

## Author Contributions

CS, LA, KL, JL, MK, and CH participated in the design of this study. CS, LA, and AS carried out the study and performed the statistical analysis. CS drafted the manuscript. The manuscript has been seen and approved by all authors. All authors confirm that they have made significant contributions to the conception or design of the work, to the analysis or interpretation of the data, to the preparation or critical revision of the work. They also checked the submitted version and gave final approval of the version to be published. All authors are responsible for all aspects of the work.

## Conflict of Interest

The authors declare that the research was conducted in the absence of any commercial or financial relationships that could be construed as a potential conflict of interest.
